# A Comprehensive Patient-Centric Analysis of Disease Burden, Treatment Challenges, and Unmet Needs in Behçet’s Disease: Insights from a Large Cohort Study

**DOI:** 10.3390/medicina62010220

**Published:** 2026-01-21

**Authors:** Samar Tharwat, Ibrahim Moustafa I. A. Abdalla, Marwa A. F. Elhefnawi, Ahmed M. M. Abutaleb, Dana M. Zein, Alia A. I. Abdelmaksoud, Rawan S. Elmetwalli, Hana M. Elkilany, Rolan M. M. Abdelaziz, Mohammed Kamal Nassar

**Affiliations:** 1 Rheumatology & Immunology Unit, Department of Internal Medicine, Faculty of Medicine, Mansoura University, Mansoura 35511, Egypt; 2 Department of Internal Medicine, Faculty of Medicine, Horus University, New Damietta 34517, Egypt; m_kamal@mans.edu.eg; 3 Medical Intern, Mansoura Manchester Medical Programme, Faculty of Medicine, Mansoura University, Mansoura 35511, Egypt; ibrahim.abdalla1920@gmail.com (I.M.I.A.A.); marwaalaaed@gmail.com (M.A.F.E.); a7medmed7at.301@gmail.com (A.M.M.A.); zeindana2@gmail.com (D.M.Z.); aliaashraf239@gmail.com (A.A.I.A.); rawanelmetwalli29@outlook.com (R.S.E.); hanamahmoud2092001@gmail.com (H.M.E.); rolanabdelaziz@gmail.com (R.M.M.A.); 4 Mansoura Nephrology and Dialysis Unit (MNDU), Department of Internal Medicine, Faculty of Medicine, Mansoura University, Mansoura 35511, Egypt

**Keywords:** Behçet’s syndrome, disease burden, patient-reported outcomes, unmet needs, treatment patterns

## Abstract

*Background and Objectives*: Behçet’s disease (BD) is a multisystem inflammatory disorder with significant physical, psychological, and social burdens. However, patient-reported outcomes and subjective symptom experiences remain under-recognized in clinical practice. This study aimed to provide a patient-centric analysis of the disease burden, treatment challenges, and unmet needs in BD. *Materials and Methods*: A multinational cross-sectional study was conducted using a structured questionnaire among 528 BD patients recruited from online support groups and a specialized clinic. The questionnaire gathered information about participants’ backgrounds, medical histories, how symptoms affected them, psychological and social factors, side effects of treatments, and their suggestions for better care. Data were analyzed descriptively. *Results*: The mean age of the participants was 41.4 years, and 69.3% were male. The most common symptoms that significantly affected daily life were severe fatigue (82.8%), joint pain and swelling (79.0%), and neurological issues (74.1%). Nearly half of patients perceived that fatigue (49.1%) and neurological symptoms (45.1%) were underestimated by healthcare providers. Psychological distress was prevalent, with 74.1% of participants reporting either depression or anxiety. Side effects related to treatment were frequently encountered (56.3%), resulting in treatment discontinuation for 53.4% of the individuals. The main unmet needs identified were fatigue reduction (59.1%), pain management (43.0%), and the minimization of side effects (59.1%). Furthermore, patients expressed a desire for enhanced communication (62.9%), validation of their unobserved symptoms (74.1%), and comprehensive disease education (67.6%). *Conclusions*: BD imposes a profound multidimensional burden, with a significant disconnect between patient experiences and their perception of clinical recognition. Fatigue, pain, psychological distress, and treatment-related challenges contribute substantially to unmet needs. A patient-centered approach emphasizing communication, symptom validation, and holistic support is essential to improving care and quality of life in BD.

## 1. Introduction

Behçet’s disease (BD) is a chronic multisystem inflammatory disorder that is characterized by the classic triad of recurrent oral ulcers, genital ulcers, and uveitis [1]. Additionally, cutaneous manifestations such as erythema nodosum-like lesions, papulopustular lesions, and pathergy are frequently observed. This disease, which affects multiple systems, can impact several organs, including the eyes, skin, joints, gastrointestinal system, and central nervous system [2]. BD primarily strikes young adults, typically those in their twenties to forties. The disease’s prevalence is particularly pronounced along the ancient Silk Road. Turkey, for instance, reports a staggering 420 cases per 100,000 individuals, a stark contrast to the significantly lower rates observed in North America and Europe [3]. The pathophysiology of Behçet’s disease (BD) involves an autoimmune and autoinflammatory process. This is characterized by an immune system imbalance that leads to increased neutrophil activity, inflammation driven by T-cells, and higher levels of pro-inflammatory cytokines. These factors together cause vasculitis, which affects blood vessels of different sizes [4].

Beyond its characteristic clinical manifestations, BD significantly affects patients’ everyday activities, substantially impairing physical functioning. This is largely attributable to enduring symptoms such as severe fatigue, widespread pain, and neurological impairments [5]. These manifestations often lead to reduced physical function and difficulties in performing everyday activities [6]. Psychological conditions, especially depression and anxiety, are frequently observed in individuals with BD [7]. Moreover, the disease precipitates considerable social and occupational limitations, with numerous patients experiencing work disruptions, hindered professional progress, and financial strain due to fluctuating disease activity and disability [8]. This multifaceted impact underscores the necessity of holistic care, which should encompass not only the management of physical symptoms but also the provision of mental health resources and social support, thereby enhancing the health-related quality of life (HRQoL) for those affected by BD [9].

BD management currently involves a range of pharmacological interventions. Colchicine is frequently employed to address mucocutaneous and articular manifestations. Corticosteroids are utilized for acute exacerbations and instances of severe organ involvement. Immunosuppressants, including azathioprine and cyclosporine, are indicated for more severe manifestations, such as ocular involvement. Apremilast is prescribed for oral ulcers, while biologics, including anti-TNF agents and interleukin inhibitors, are reserved for refractory cases [10]. Moreover, the adverse effects associated with these medications significantly impact patients, often necessitating limitations on the duration and intensity of treatment. Accessibility issues and the high cost of biologic drugs further impede the provision of effective care for numerous patient [11]. Consequently, these factors underscore the ongoing challenges in achieving sustained disease control and improving patient HRQoL [12].

While clinical tools remain important for monitoring BD activity, they frequently fail to capture the full range of the patient’s experience [9]. Symptoms including fatigue, cognitive impairment, and pain are frequently “invisible” to physicians and may be underestimated [13]. As a result, patient-reported outcomes (PROs) are essential for the provision of patient-centered, comprehensive care, as they provide direct insights into patients’ symptoms, unmet needs, and treatment satisfaction. Nevertheless, the integration of PROs into routine clinical practice for Behçet’s disease is still inconsistent, indicating a substantial gap that this research seeks to address by underscoring the critical significance of incorporating the patient’s voice in disease management and therapeutic decision-making.

This study aimed to bridge the gap between clinical evaluation and patients’ lived experiences with Behçet’s illness by systematically studying the prevalence, intensity, and significance of invisible symptoms such as fatigue, cognitive dysfunction, and pain. It also aimed to evaluate PROs linked to unmet requirements and treatment satisfaction, so providing a holistic knowledge of the disease burden from the patient’s perspective.

## 2. Materials and Methods

### 2.1. Study Design and Setting

This cross-sectional analytical study was conducted on 528 individuals diagnosed with BD from different countries during the period between March to August 2025. Online BD patient and support groups were used to find participants, and private messages, emails, or WhatsApp were used to get in touch. Patients followed at the BD clinic at Mansoura University’s Faculty of Medicine were also included. Every participant completed the inclusion requirements of being older than 18 years and having a verified diagnosis of BD from a rheumatologist according to internationally recognized diagnostic criteria (International Criteria for Behçet’s Disease, ICBD) [14]. Individuals with concurrent autoimmune diseases or psychiatric disorders were excluded. Before beginning the questionnaire, participants were presented with an introductory section detailing the study’s purpose and objectives and were required to provide informed consent by signing prior to proceeding with the survey. Participants’ anonymity and data confidentiality were rigorously preserved.

### 2.2. Sample Size Calculation

We determined the appropriate sample size for this study using the online sample size calculator RaoSoft^®^ (Seattle, WA, USA, 2004); a conservative estimate was based on a 50% response rate, a 5% margin of error, and a 95% confidence level. The minimum required sample size was 377 participants, but the study included 528 participants, surpassing this threshold.

### 2.3. Ethical Consideration

This study was conducted in compliance with the Helsinki Declaration [15], and the Institutional Research Board of Mansoura University’s Faculty of Medicine granted its approval to the study protocol (approval registration number: R.25.12.3496, approval date: 22 June 2025).

### 2.4. Questionnaire Structure

The questionnaire was created after conducting a thorough review of the current literature on BD. The instrument, written in English, mainly used multiple-choice questions to gather demographic, clinical, and patient-reported outcome data. A panel of five rheumatology specialists evaluated the first version of the questionnaire, offering feedback on its content validity. The pilot study then included a group of thirty individuals diagnosed with BD, representing a wide range of ages and backgrounds.

The purpose of this pilot phase was to assess the questionnaire’s length, organization, and clarity as well as to obtain participant feedback on how comprehensible it was overall. A few minor adjustments were made in response to the feedback. Cronbach’s alpha was used to assess the internal consistency of the finalized questionnaire, and the value of 0.87 indicated good reliability. The pilot participants’ data was omitted from the main study’s final statistical analysis.

#### 2.4.1. Participant Demographics

Participants’ sociodemographic information was gathered through the questionnaire. This data included age, gender, marital status, and educational background.

#### 2.4.2. Clinical Characteristics and Treatments

This section outlined the clinical profiles and treatment history of the participants. It included inquiries about the duration of the disease since diagnosis, the frequency and nature of flare-ups, and the presence of cumulative clinical manifestations such as oral and genital ulcers, skin lesions, joint involvement, ocular symptoms, neurological and gastrointestinal problems, and vascular involvement (e.g., deep vein thrombosis, arterial aneurysms, or other vasculitic events). Data regarding the familial history of BD was also gathered. Furthermore, the questionnaire evaluated the therapy methods administered to patients, encompassing both medical therapies and non-medical interventions.

#### 2.4.3. Impact on Daily Activities and Perceptions

Participants were inquired about diverse manifestations, including oral and genital ulcers, dermal lesions, arthralgia, ocular inflammation, neurological and gastrointestinal symptoms, alongside less apparent symptoms such as fatigue, depression, and anxiety. They were also asked to identify which symptoms they believed substantially impacted their daily lives and which they perceived as undervalued or underestimated by the medical team.

#### 2.4.4. Life Activities Affected

This section outlines the everyday living characteristics affected by Behçet’s disease as stated by the study participants. The questionnaire comprised items evaluating multiple dimensions of daily activities, including mobility, self-care, social connections, and occupational involvement.

#### 2.4.5. Quality of Life and Psychosocial Factors

This section of the questionnaire included items evaluating the overall quality of life and several psychosocial dimensions encountered by patients with Behçet’s disease. The inquiries examined psychological symptoms including depression and anxiety, felt stigma, emotions of misfortune related to the disease, and social challenges encompassing difficulties in travel, moving, and communication.

#### 2.4.6. Treatment Side Effects and Unmet Needs

This section encompassed various questions designed to evaluate the prevalence and kind of treatment-related adverse effects experienced by individuals with BD. Participants were inquired about prevalent side effects including gastrointestinal problems, weight gain, mood alterations, heightened infection susceptibility, and other systemic complications. Furthermore, inquiries examined patients’ unaddressed treatment requirements, particularly in domains where existing medicines are inadequate, like fatigue alleviation and pain control.

#### 2.4.7. Suggestions for Treatment Improvement

This section compiled patient insights regarding potential enhancements in treatment and care. Multiple-choice questions on a variety of topics were included in the assessment, such as communication with healthcare providers, the frequency of medical appointments, the availability of non-medical interventions (such as physical therapy and psychological support), educational initiatives on disease management, medication availability, and cost reduction.

### 2.5. Statistical Analysis

Statistical analysis was performed using SPSS version 23.0 (IBM Corp., Armonk, NY, USA). Continuous variables were assessed for normality using the Shapiro–Wilk test and reported as mean ± standard deviation (SD) for normally distributed data. Categorical variables were expressed as frequencies and percentages.

## 3. Results

Table 1 summarizes the sociodemographic characteristics of the 528 patients with Behçet’s disease included in the study. The mean age was 41.42 years (±12.55), with a predominance of males (69.3%). Most participants were single (63.3%), and 68.8% reported having children.

Table 2 summarizes the clinical characteristics and treatment modalities among the study BD patients. The majority had disease duration under 10 years, with common manifestations including mouth ulcers (91.1%), skin lesions (89%), and genital ulcers (71.6%). Frequent disease flare-ups and persistent symptoms were reported by most patients. Over half received medical treatments such as corticosteroids and biologics.

Table 3 highlights the symptoms of Behçet’s disease that most affect patients’ daily lives and those that patients perceived as being under-rated by their medical team. The most impactful symptoms included severe fatigue (82.8%), joint pain or swelling (79.0%), neurological symptoms (74.1%), and mouth ulcers (64.4%). A substantial proportion of patients felt that symptoms like fatigue (49.1%), neurological symptoms (45.1%), and gastrointestinal symptoms (32.2%) were perceived as underestimated by healthcare providers.

**Figure 1:**
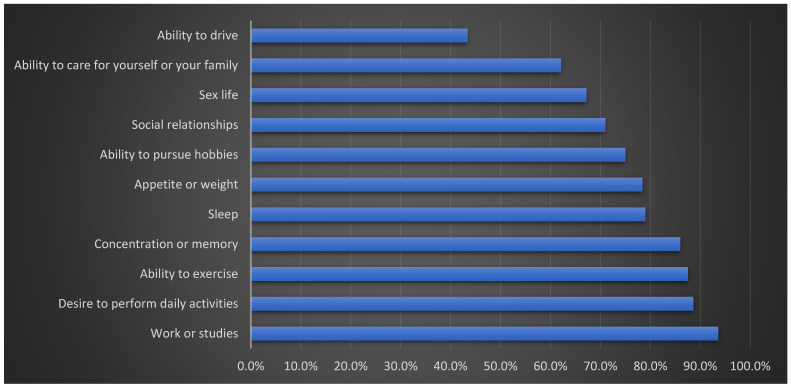
Life activities affected by the disease in the Behcet’s disease patients studied (n = 528).

Figure 1 illustrates the various daily activities impacted by BD among the studied BD patients. The data indicate that a majority of patients experienced difficulties in key life domains, including mobility, social interactions, and routine tasks.

Table 4 summarizes the psychosocial impact and quality of life among the 528 Behçet’s disease patients. A significant proportion reported psychological symptoms, with 74.1% experiencing depression or anxiety and 63.3% expressing a need for psychological support. Nearly half felt stigmatized (45.0%) or unlucky (49.0%) due to their condition. 

Table 5 summarizes the treatment-related adverse effects and unmet needs among the 528 BD patients with comparison between glucocorticoid users and non-users. Over half (56.3%) reported experiencing side effects, including gastrointestinal issues (42.2%), headaches (64.6%), mood disturbances (47.9%), and weakness or osteoporosis (53.2%). Treatment discontinuation due to side effects was reported by 53.4%. Many patients faced barriers to treatment access (43.8%) and financial challenges (43.9%). A majority (74.2%) identified unmet treatment needs, notably reducing fatigue (59.1%), pain management (43.0%), minimizing side effects (59.1%), and improving quality of life (39.6%). 

Table 6 summarizes patient-reported suggestions for improving treatment and care. The most frequently suggested improvements included listening more to patient complaints and invisible symptoms (74.1%), providing educational programs about disease and coping strategies (67.6%), and enhancing communication with the medical team (62.9%).

## 4. Discussion

This large, multinational cohort study provides a comprehensive patient-centric evaluation of the profound burden of BD, highlighting a significant disconnect between patient experiences and clinical recognition. Our principal findings reveal that the most impactful symptoms on patients’ daily lives—severe fatigue, joint pain, and neurological manifestations—are frequently perceived by patients as being underestimated by healthcare providers. Furthermore, the study highlights a significant prevalence of psychological distress, along with considerable social and vocational limitations. These issues are made worse by challenges related to treatment, such as a high rate of side effects and difficulties in accessing care. The majority of patients reported unmet needs, especially regarding fatigue and pain management. Their main suggestions for improvement focused on better communication, more recognition of their reported symptoms, and more educational support.

Our findings, derived from descriptive analyses, highlight the patient-reported burden and unmet needs in Behçet’s disease. While these analyses do not establish causality, they provide valuable cross-sectional insights into symptom prevalence, treatment challenges, and patient perceptions. The clinical and therapeutic profile of the BD patients in this study largely aligns with the established multisystem nature of the disease. Most patients exhibited classical manifestations, including high frequencies of oral ulcers (91.1%), skin lesions (89%), and genital ulcers (71.6%), consistent with prevalence data reported by Gheita et al. (2019) [16] and Lavalle et al. (2024) [2], who emphasized mucocutaneous involvement as a hallmark of BD across diverse populations. Therapeutically, a significant proportion of patients received corticosteroids and immunosuppressants, including azathioprine and cyclosporine, confirming the reliance on these agents for controlling systemic inflammation and preventing severe organ involvement, as supported by Fazaa et al. (2024) [3] and Alibaz-Oner and Direskeneli (2021) [11]. Biologic therapies, particularly anti-TNF agents, were utilized in refractory cases, highlighting advances in targeted treatment options but also underscoring challenges related to accessibility and cost noted by Senusi et al. (2022) [12].

In this study, the symptoms most profoundly affecting the daily lives of BD patients were severe fatigue, joint pain or swelling, neurological symptoms, and recurrent oral ulcers. These findings align well with those reported by Ilhan et al. (2018) [13] and Tharwat et al. (2025) [5], who highlighted fatigue and musculoskeletal manifestations as principal contributors to impaired physical functioning and reduced quality of life in BD cohorts. Fatigue, reported by over 80% of our participants as a major daily life disruptor, concurs with global evidence emphasizing its prevalence and significant association with disease activity, depression, and disability. Oral ulcers, although a hallmark symptom, were ranked lower in impact compared to less visible symptoms, which may be underestimated in clinical practice, as the present study’s participants indicated. Psychological burdens such as depression and anxiety, reported by over 70% of patients, further compound the symptom burden, contributing to social and occupational difficulties, consistent with the data by El Hasbani et al. (2022) [7].

A significant finding from this study is the identification of symptoms that BD patients reported as being under-recognized by their medical teams. Among the 528 patients studied, neurological symptoms (45.1%) and severe fatigue or exhaustion (49.1%) were predominantly reported as being under-recognized. Additionally, depression or anxiety (30.7%) and sleep disturbances (29.5%) also ranked high among symptoms perceived as overlooked. These findings align with similar patient-reported outcome studies by Ramadan et al. (2025) [9], which documented the frequent invisibility of fatigue and neuropsychiatric symptoms in clinical assessments despite their profound impact on daily living. The substantial patient-reported perception of fatigue and psychological distress as under-rated resonates with previous observations [17], emphasizing the need to integrate mental health evaluation and supportive care into routine clinical practice. Gastrointestinal involvement similarly presents a challenge, often overshadowed by more overt mucocutaneous or ocular manifestations [18]. This discordance between patient experience and clinical recognition underscores the necessity for improved communication and comprehensive symptom evaluation, advocating for multidisciplinary approaches and patient-centered models that prioritize both objective disease control and subjective symptom relief.

The findings show that 74.1% of our BD patients reported depression or anxiety and that nearly half felt stigmatized or unlucky due to their condition underscore the profound psychosocial burden imposed by BD. These patient-reported rates are notably higher than those described in many chronic inflammatory diseases and align with previous studies showing that more than half of BD patients experience significant mental health challenges [19,20]. Ramadan et al. (2025) reported depression prevalence exceeding 50% and anxiety rates around 60% in BD, highlighting frequent under-recognition of these problems in clinical care [9]. In a qualitative study by Jahani et al. (2024), patients described deep emotional and communicative instability, isolation, and a pervasive sense of despair shaped by unpredictable symptoms and the fear of disease complications [21]. Stigmatization and negative self-perception, observed in nearly half of this study’s participants, were also salient in recent multinational surveys, where BD’s visible and invisible symptoms contributed to social withdrawal, reluctance to seek support, and decreased motivation [22,23].

In the current study, the high prevalence of treatment-related adverse effects and unmet needs among BD patients in this study is striking: 56.3% experienced side effects; including gastrointestinal issues (42.2%), headaches (64.6%), mood disturbances (47.9%), and over half discontinued treatment due to intolerance. Such figures parallel recent reports: Ramadan et al. (2025) [9] and Sulu et al. (2024) [24] highlight that side effects, particularly those affecting the gastrointestinal and nervous systems, are among the leading reasons for poor adherence or treatment discontinuation in BD cohorts, with rates frequently exceeding 50%. Additionally, barriers to therapy, including financial constraints (43.9%) and access difficulties (43.8%), remain a global challenge for individuals with BD, as echoed by Di Cianni et al. (2024), who emphasized substantial health system and out-of-pocket burdens associated with multi-drug regimens and regular follow-up [25].Most notably, more than 70% of patients identified unmet treatment needs, pointing to persistent fatigue, pain, side effects, and the desire for improved quality of life even when medically managed. Patient experience research reiterate that standard immunosuppressive or biologic therapies alone are rarely adequate to restore full functional status or well-being in BD, and that holistic, individualized, and multidisciplinary care strategies, including pain management, patient education, and integrated psychological support, are key to addressing the residual burden [26,27,28].

The present study highlights critical patient-reported suggestions for enhancing treatment and care in BD, with the top priorities including greater attention to patient complaints and invisible symptoms (74.1%), educational programs about the disease and coping strategies (67.6%), and improved communication with healthcare teams (62.9%). These findings resonate with previous literature emphasizing the need for patient-centered approaches in chronic disease management [29,30]. BD patients often feel unheard regarding their subjective symptoms such as fatigue and neurological complaints, which are frequently under-recognized in clinical practice [2]. The importance of education programs aligns with evidence that increased patient knowledge enhances self-management, treatment adherence, and psychological well-being in Behcet’s and other chronic illnesses. Furthermore, consistent with Jahani et al. (2024), effective communication and extended consultation times are crucial for building trust, addressing complex symptomatology, and supporting psychosocial needs [21].

This study benefits from a large, well-characterized cohort of 528 Behçet’s disease patients, providing robust patient-centered insights into the multifaceted burden of the disease across physical, psychological, social, and treatment-related domains. The inclusion of detailed patient-reported outcomes enhances understanding of symptom impact and unmet needs that are often overlooked in clinical assessments. However, limitations include the cross-sectional design, which precludes causal inferences or longitudinal evaluation of symptom trajectories and treatment effects. Potential selection bias may exist, as patients engaged in specialized clinics or willing to participate in research could differ from the broader BD population. Additionally, reliance on self-reported data introduces the possibility of recall bias or subjective interpretation. Despite these limitations, the comprehensive scope and patient-centric focus provide valuable guidance for improving multidisciplinary care models and highlight areas requiring further prospective research.

Future research should focus on longitudinal studies to capture the dynamic nature of BD symptoms and treatment effects over time. Investigations into personalized, multidisciplinary interventions that address both physical and psychosocial needs are needed. Exploring patient coping strategies, stigma experiences, and adherence factors through qualitative approaches will deepen understanding of patient challenges. Additionally, evaluating emerging therapies and validating digital health tools to monitor patient-reported outcomes can enhance care delivery and improve quality of life.

## 5. Conclusions

In conclusion, this study provides comprehensive patient-centered insight into the significant burden of Behçet’s disease, highlighting that severe fatigue, joint pain, neurological symptoms, and psychological distress profoundly affect daily functioning and quality of life. The findings reveal a critical gap between patient experiences and their perception of clinical recognition, with many symptoms reported as under-recognized by patients. Treatment-related adverse effects and barriers to access further contribute to unmet needs. Patients expressed a strong need for improved communication, validation of invisible symptoms, and enhanced education and support.

## Figures and Tables

**Table 1:** Sociodemographic Data of the Behcet’s disease patients studied (n = 528).

Variable Mean ± SD, n (%)	Behcet’s Disease Patients (n = 528)
Age	41.42 ± 12.55
Gender	
Female	366 (69.3)
Male	162 (30.7)
Marital Status	
Single	135 (25.6)
Married	334 (63.3)
Divorced	48 (9.1)
Widowed	11 (2.1)
Have children	363 (68.8)
Education Level	
Less than high school	31 (5.9)
High school	150 (28.4)
University	253 (47.9)
Postgraduate	94 (17.8)

**Table 2:** Clinical and therapeutic data of the Behcet’s disease patients studied (n = 528).

Variable n (%)	Behcet’s Disease Patients (n = 528)
Number of years since Behçet’s disease diagnosis	
Less than 1 year	79 (15.0)
1–5 years	190 (36.0)
6–10 years	87 (16.5)
More than 10 years	172 (32.6)
Cumulative manifestations	
Mouth ulcers	481 (91.1)
Genital ulcers	378 (71.6)
Skin rash or acne-like lesions	424 (80.3)
Joint pain or swelling	470 (89.0)
Eye inflammation or pain (uveitis/iritis)	339 (64.2)
Neurological symptoms	418 (79.2)
Gastrointestinal symptoms	392 (74.2)
Vascular involvement	121 (22.9)
Family History of Behçet’s disease	55 (10.4)
Flare-ups in the past 5 years	
None	17 (3.2)
Once	37 (7.0)
2–5 times	176 (33.3)
More than 5 times	298 (56.4)
Persistent Symptoms Between Flare-ups	428 (81.1)
Treatment Type	
Medical treatment	402 (76.1)
Non-medical treatment (physical, psychological, etc.)	20 (3.8)
Home treatment	39 (7.4)
No treatment	25 (4.7)
Medical treatment	
Corticosteroids	266 (50.4)
Azathioprine	155 (29.4)
Methotrexate	53 (10.0)
Cyclosporine	18 (3.4)
Cyclophosphamide	8 (1.5)
Infliximab	55 (10.4)
Adalimumab	71 (13.4)
Etanercept	10 (1.9)
Colchicine	337 (63.8)
Apremilast	32 (6.1)
Interferon alpha	6 (1.1)
Dapsone	13 (2.5)
Pentoxifylline	13 (2.5)
Tocilizumab	1 (0.2)

**Table 3:** Symptoms that most affect daily life and symptoms that are perceived by the Behcet’s disease patients as under-rated by the medical team (n = 528).

Variable n (%)	Affect Daily Life (n = 528)	Perceived as Under-rated (n = 528)
Mouth ulcers	340 (64.4)	108 (20.5)
Genital ulcers	213 (40.3)	106 (20.1)
Skin rash or acne-like lesions	237 (44.9)	107 (20.3)
Joint pain or swelling	417 (79.0)	164 (31.1)
Eye inflammation or pain (uveitis/iritis)	224 (42.4)	74 (14.0)
Blurred or impaired vision	276 (52.3)	92 (17.4)
Photophobia	261 (49.4)	82 (15.5)
Neurological symptoms	391 (74.1)	238 (45.1)
Gastrointestinal symptoms	335 (63.4)	170 (32.2)
Vascular involvement	115 (21.8)	60 (11.4)
Chest pain or shortness of breath	194 (36.7)	103 (19.5)
Fever	112 (21.2)	46 (8.7)
Severe fatigue or exhaustion	437 (82.8)	259 (49.1)
Hair loss	204 (38.6)	84 (15.9)
Depression or anxiety	337 (63.8)	162 (30.7)
Sleep disturbances	357 (67.6)	156 (29.5)
Difficulty eating or drinking due to mouth ulcers	268 (50.8)	80 (15.2)
Difficulty walking or moving due to joint or nerve pain	350 (66.3)	163 (30.9)
Difficulty with social interaction due to visible symptoms or pain	256 (48.5)	115 (21.8)

**Table 4:** Quality of life and psychosocial impact among the Behcet’s disease patients studied (n = 528).

Variable Mean ± SD, n (%)	Behcet’s Disease Patients (n = 528)
Overall quality of life	5.05 ± 2.17
Experience psychological symptoms such as depression or anxiety	391 (74.1)
Need psychological support	334 (63.3)
Feel stigmatized because of the disease	240 (45.5)
Feel unlucky because of the disease	259 (49.1)
Have difficulty traveling or moving around because of the disease	346 (65.5)
Have difficulty communicating with others	190 (36.0)

**Table 5:** Treatment side effects and unmet needs among the Behcet’s disease patients studied (n = 528) with comparison between glucocorticoid users and non-users.

Variable Mean ± SD, n (%)	Total Behcet’s Disease Patients (n = 528)	Glucocorticoid non-users (n = 266)	Glucocorticoid Users (n = 262)	*p*
Treatment Side Effects				
Gastrointestinal disturbances	297 (56.3)	138 (52.7)	159 (59.8)	0.1
Weight gain	223 (42.2)	95 (36.3)	128 (48.1)	0.006 *
High blood pressure	95 (18.0)	34 (13.0)	61 (22.9)	0.003 *
Mood swings, depression, or anxiety	257 (48.7)	110 (42.0)	147 (55.3)	0.002 *
Increased risk of infection	253 (47.9)	121 (46.2)	132 (49.6)	0.429
Weakness or osteoporosis	199 (37.7)	84 (32.1)	115 (43.2)	0.008 *
Muscle pain	281 (53.2)	129 (49.2)	152 (57.1)	0.069
Blood disorders	120 (22.7)	51 (19.5)	69 (25.9)	0.076
Liver or kidney dysfunction	99 (18.8)	43 (16.4)	56 (21.1)	0.172
High blood sugar or onset of diabetes	79 (15.0)	28 (10.7)	51 (19.2)	0.006 *
Hair loss	181 (34.3)	93 (35.5)	88 (33.1)	0.559
Increased eye pressure or glaucoma	76 (14.4)	29 (11.1)	47 (17.7)	0.031 *
Cataract	66 (12.5)	27 (10.3)	39 (14.7)	0.13
Drug allergy	81 (15.3)	36 (13.7)	45 (16.9)	0.311
Injection site reactions	83 (15.7)	37 (14.1)	37 (14.1)	0.317
Headache	234 (44.3)	113 (43.1)	121 (45.5)	0.585
General fatigue or severe exhaustion	341 (64.6)	164 (62.6)	177 (66.5)	0.343
Upper respiratory infections	156 (29.5)	75 (28.6)	81 (30.5)	0.646
Sleep disturbances	267 (50.6)	119 (45.4)	148 (55.6)	0.019
Difficulties in Accessing Treatment	231 (43.8)	113 (43.1)	118 (44.4)	0.776
Financial Barriers to Treatment	232 (43.9)	102 (38.9)	130 (48.9)	0.021 *
Medication Discontinuation Due to Side Effects	282 (53.4)	145 (55.3)	137 (51.5)	0.377
Treatment Satisfaction				
Yes	147 (27.8)	75 (28.6)	72 (27.1)	0.727
No	132 (25.0)	68 (26.0)	64 (24.1)	
Somewhat	249 (47.2)	119 (45.4)	130 (48.9)	
Unmet Treatment Needs				
Reducing fatigue	392 (74.2)	187 (71.4)	205 (77.1)	0.135
Improving pain	312 (59.1)	154 (58.8)	158 (59.4)	0.885
Reducing side effects	227 (43.0)	93 (35.5)	134 (50.4)	0.001 *
Improving quality of life	312 (59.1)	151 (57.6)	161 (60.5)	0.499
Psychological support	209 (39.6)	89 (34.0)	120 (45.1)	0.009 *
Social support	183 (34.7)	86 (32.8)	97 (36.5)	0.379
Non-medical treatment	170 (32.2)	79 (30.2)	91 (34.2)	0.318

* *p* < 0.05.

**Table 6:** Patient-reported treatment improvement suggestions among the Behcet’s disease patients studied (n = 528).

Variable n (%)	Behcet’s Disease Patients (n = 528)
Improving communication with the medical team	332 (62.9)
Increasing the number of visits or continuous follow-up	259 (49.1)
Providing non-medical treatments (e.g., psychological support, physical therapy, etc.)	321 (60.8)
Listening more to patient complaints and invisible symptoms	391 (74.1)
Providing educational programs about disease and coping strategies	357 (67.6)
Facilitating access to medications	288 (54.5)
Reducing treatment costs	306 (58.0)
Reducing medication side effects	308 (58.3)
Providing patient support programs (support groups, workshops, etc.)	329 (62.3)
Providing digital applications or tools for health monitoring	301 (57.0)
Allowing more time during medical visits	269 (50.9)

## Data Availability

The datasets generated during and/or analyzed during the current study are available from the corresponding author on reasonable request.

## Abbreviations

BD	Behçet’s Disease
HRQoL	Health-Related Quality of Life
PROs	Patient-Reported Outcomes
SD	Standard Deviation
OR	Odds Ratio
TNF	Tumor Necrosis Factor
